# *In situ* detection and mass spectrometry imaging of protein-related metabolites in Bombyx batryticatus before and after frying with wheat bran

**DOI:** 10.3389/fpls.2023.1144556

**Published:** 2023-04-06

**Authors:** Pai Liu, Jie-Min Wang, Hao-Chuan Guo, Meng-Wei Zhao, Yong-Xing Song, Hui Guo, Xu-Hong Duan, Yu-Ping Yan, Yu-Guang Zheng

**Affiliations:** ^1^ College of Pharmacy, Hebei University of Chinese Medicine, Shijiazhuang, Hebei, China; ^2^ Traditional Chinese Medicine Processing Technology Inheritance Base of the State Administration of Traditional Chinese Medicine, College of Pharmacy, Hebei University of Chinese Medicine, Shijiazhuang, Hebei, China

**Keywords:** MALDS-MSI, before and after frying, OPLS-DA, protein-related metabolites, Chinese medicine, Bombyx batryticatus

## Abstract

Bombyx batryticatus is derived from the dried larva of *Bombyx mori* Linnaeus infected by *Beauveria bassiana* (Bals.) Vuillant. Raw Bombyx batryticatus should be stir-fried before oral administration due to its irritation to the gastrointestinal tract. Nevertheless, it is still an arduous task to uncover the intrinsic mechanism of Bombyx batryticatus processing. In this study, we collected two types of Bombyx batryticatus, one being stir-fried and the other serving as a control. Then, an informative approach, which integrated matrix-assisted laser desorption/ionization mass spectrometry imaging (MALDI-MSI) with chemometrics analysis, was established to screen processing-associated markers and reveal *in situ* spatial distribution patterns of protein-related metabolites. After optimization of experimental conditions, 21 ions were initially detected from Bombyx batryticatus, including amino acids and peptides. In addition, 15 differential markers were screened by orthogonal projection to potential structure discriminant analysis (OPLS-DA), which were localized and visualized in the transverse section of Bombyx batryticatus by MSI. Eventually, it can be demonstrated that the stir-frying process reduces toxicity while potentially boosting specific biological activities of Bombyx batryticatus. In summary, the established strategy could not only clarify the chemical transformation of protein-related metabolites from Bombyx batryticatus before and after frying with wheat bran, but also reveal the significance of Chinese medicine processing technology.

## Introduction

**Figure 1:**
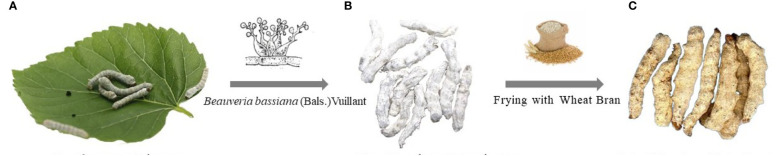
The formation of raw and fried Bombyx batryticatus. **(A)** Bombyx mori Linnaeus and mulberry leaves. **(B)** Raw Bombyx batryticatus. **(C)** Fried Bombyx batryticatus.

Bombyx batryticatus is a famous Chinese medicine, which belongs to the dry bodies of the 4th and the 5th instar larvae of *Bombyx mori* Linnaeus infected (or artificially inoculated) with *Beauveria bassiana* (Bals.) Vuillant (Chinese Pharmacopoeia Commission, 2020). Bombyx batryticatus has a long history of clinical application in Chinese medicine, which was first recorded in the *Classic of Shennong Materia*. The main efficacy of Bombyx batryticatus is resolving phlegm and dispersing blood stasis, dispelling wind and settling shock. It is mainly used in the treatment of liver wind and phlegm, convulsion, infantile shock, tetanus, sore throat, and rubella pruritus (Wang et al., 2021a). The first-class Bombyx batryticatus has a hard texture, thick strips, white color and bright cross-section (Hu, 2017). Beauveria Batryticatus contains various chemical components, such as protein, amino acid, ammonium oxalate, enzymes, nucleoside bases, trace elements, etc. (Bai et al., 2018). The *Bombyx mori* feeds mainly on leaves of mulberry (*Morus alba* Linn.) throughout their life, absorbing, accumulating and metabolizing a variety of chemicals within their bodies. After invading the *Bombyx mori*, *Beauveria bassiana* proceed to multiply and grow based on the larva as a resource of nutrients, which was essentially a fermentation process by the fungus. Furthermore, the original components can be transformed into new components with higher activity in the form of hydroxylation, redox reaction, sulfonation, and hydrolysis of epoxide 4-O-methylglucoside (Xing et al., 2019). Accordingly, the bioactivities of Bombyx batryticatus are essentially derived from the co-action of *Bombyx mori*, mulberry leaves and *Beauveria bassiana* (**Figure 1**).

The processing of Chinese medicine is a unique pharmaceutical technology in China, which refers to the manufacturing process of stir-frying, roasting, carbonizing, steaming, etc., aiming to enhance efficacy and reduce toxicity (Li et al., 2020). There are two types of Bombyx batryticatus widely used in clinical applications, namely raw and fried forms. Bombyx batryticatus research has always focused on small molecular compounds with little protein-related metabolites involved in the past. For example, Wang et al. (2022) established a high performance liquid chromatography (HPLC) fingerprint combined with chemical pattern recognition technology to evaluate the quality of Bombyx batryticatus. A liquid chromatography-tandem mass spectrometry (LC-MS/MS) technology was used to qualitatively analyze the chemical constituents in water-soluble substances of Bombyx batryticatus (Wang et al., 2021b). Although several attempts have been made to elucidate the active metabolites in Bombyx batryticatus, the intrinsic transformation mechanisms of protein-related metabolites, which account for the largest proportion of endogenous components, from Bombyx batryticatus before and after frying are still unclear. Chinese medicine is a complex mixture with hundreds of chemical components. the active ingredients can be easily altered or lost, and the spatial distribution of various components in tissues is also severely damaged during processing, such as extraction, purification and enrichment, making it impossible to achieve *in situ* analysis of active ingredients (Wang et al., 2016). In recent years, matrix-assisted laser desorption/ionization mass spectrometry imaging (MALDI-MSI) has become an imaging technique for studying the composition and distribution of molecules or ions at the tissue or cellular level (Li et al., 2020), which is characterized with high sensitivity, high spatial resolution and high laser scanning speed (Zemaitis et al., 2022). Shu et al. (2010) performed MSI to confirm that ginsenosides were located more in the cortex and periderm than in the medulla of a lateral root of *Panax ginseng*. Li et al. (2021a) used MALDI-MSI combined with multiple matrixes to analyze spatial metabolomes on root sections of the two *Paeonia* species. Li et al. (2014) showed the distribution of major tissue-specific metabolites in the rhizome of *Glycyrrhiza glabra* (licorice) at cellular level by atmospheric pressure MALDI-MSI. However, this technique has not been applied to study the distribution of chemical components in tissues during processing Bombyx batryticatus.

In the present work, a mass spectrometry-based profiling and imaging strategy was established to reveal the chemical transformation of protein-related metabolites from Bombyx batryticatus before and after frying with wheat bran. To begin with, the chemical profiling of peptides and amino acids from raw and fried Bombyx batryticatus was achieved by identifying characteristic ion fragments and matching our in-built database. Subsequently, multivariate statistical analysis was applied to screen processing-related markers. Finally, MALDI-MSI was performed to testify the markers discovered and visualize their chemical transformation trajectories.

## Materials and methods

### Instruments and reagents

Instruments used in this study included Bruker Rapiflex MALDI-TOF/TOF mass spectrometer equipped with the FlexAnalysis data processing workstation (Bruker, Karlsruhe, Germany), GET-Sprayer (І) (HIT Co., Ltd., Beijing, China), Indium Tin Oxide (ITO) conductive slides (diagonal resistance ~40 Ω, Bruker, Karlsruhe, Germany), vacuum desiccator (Shanghai Yueyang Electronic Technology Co., Ltd., Shanghai, China), and cryomicrotome (Leica CM1950, Nussloch, Germany).

The following reagents were used in this study: methanol and acetonitrile (mass spectrometry pure, Merck, Darmstadt, Germany), optimum cutting temperature (OCT) embedding agent (Leica, Nussloch, Germany), and trifluoroacetic acid (TFA) (Sigma, St. Louis, MO, USA). Water was purified by a Milli-Q filtration system (Millipore, Billerica, MA, USA). A total of 26 compounds representing five different classes of bioactive components (e.g., amino acids, peptides, nucleosides, flavonoids, and others) were procured from the National Institutes of Food and Drug Control (Beijing, China) and used as reference compounds (**Supplementary Table 1**).

### Sample preparation

Both raw Bombyx batryticatus (RBB) and fried Bombyx batryticatus (FBB) were purchased from Hebei Chenghai Tang Chinese Medicine Co., Shijiazhuang, Hebei Province, China. Morphological identification of Bombyx batryticatus was carried out by Professor Yu-Guang Zheng at the Processing Technology Innovation Center of Traditional Chinese Medicine (TCM), Shijiazhuang, Hebei Province. After the matrix application, three samples from the two types of Bombyx batryticatus were selected, and three biological replicates of each sample were performed. Bombyx batryticatus was placed in a freezing microtome at -25°C for 30 min and fixed to the microtome tray with OCT embedding agent. Frozen tissue sections of 20 μm in thickness were transferred to ITO conductive glass slides and vacuumed in a vacuum desiccator for 20 min.

### Matrix and standard solution preparation

200mg control powder of 2-mercaptobenzothiazole (2-MBT) was weighed and dissolved in 10 ml of methanol-water (7:3) solution, followed by the addition of 0.1% trifluoroacetic acid (TFA) dropwise to promote ionization, and finally prepared into a matrix solution with a mass concentration of 20 mg/ml. The control solution of standard substances was prepared at 1.0 mg/ml. Then, 1 μl of the control solution was added dropwise to the Bruker ground steel target plate and dried in vacuum for 20 minutes until the sample spot was dry. After that, the matrix solution (1 μl) was added dropwise to cover the sample spot and dried in vacuum again until the solvent evaporated, followed by MALDI-MS analysis.

### Matrix application

Vacuum-dried Bombyx batryticatus tissue sections were fixed in a matrix sprayer and matrix spraying was performed with 2-MBT. The spray program was performed for 8 cycles with a matrix flow rate of 0.075 mL/min, nozzle temperature at 60°C, nozzle travel speed of 800 mm/min, nozzle travel row spacing of 3 mm, and nozzle-sample row spacing of 3 cm. Tissue sections were prepared for the MALDI mass spectrometry analysis after spraying.

### MALDI-TOF-MSI

Tissue sections of sprayed matrix were placed in a Bruker Rapiflex MALDI-TOF/TOF type mass spectrometer (Bruker, Karlsruhe, Germany) equipped with a smartbeamTM 3D laser for mass spectrometry imaging analysis. The wavelength was 355 nm at a sampling rate of 2.5 GS/s. MALDI mass spectrometry imaging was performed in a positive ion detection mode with a mass-to-charge ratio (m/z) ranging from 100 to 1500 and a spatial resolution of 100 μm for scanning samples.

### MALDI-TOF-MS/MS

Peak values of precursor ions were obtained in the positive ionization and reflectron modes. Tandem mass spectrometry was performed in a LIFT mode with a mass tolerance of 0.05%. Precursor ions were accelerated at 7.5 kV under optimized ion-selective gating. The acceleration voltage of the LIFT unit was set to 19 kV. All mass spectra were analyzed with FlexAnalysis software 3.4 (https://softwaretopic.informer.com/). Default settings were used for baseline correction and smoothing.

### Data analysis

For the mass spectrometry analysis, FlexAnalysis 3.4 software of Bruker (https://softwaretopic.informer.com/) was used for initial mass spectrum observation and processing. These extracted mass spectra were baseline subtracted, normalized, and recalibrated based on the total ion count (TIC) in the observed mass range. Ion screening was performed based on accurate mass-to-charge ratios from the literature and databases of METLIN (https://metlin.scripps.edu/) and HMDB (https://hmdb.ca). Three ion addition forms, i.e., [M+H]^+^, [M+Na]^+^, and [M+K]^+^, were used to perform database search. Data sets were tabulated and imported into SIMCA 14 software (Umetrics, Malmo, Sweden) for multivariate statistical analyses, including principal component analysis (PCA), orthogonal projection to potential structure discriminant analysis (OPLS-DA) and hierarchical cluster analysis (HCA), to distinguish samples with different quality characteristics and identify potential quality-related markers. In order to further confirm the differential metabolites identified above, a Student’s t-test analysis was conducted, which will be illustrated in the form of statistical tables (**Supplementary Table 2**).

## Results

### Chemical composition profile of Bombyx batryticatus before and after frying with wheat bran

**Figure 2:**
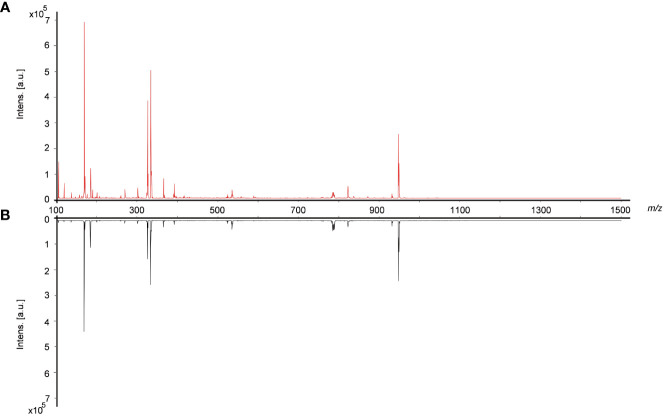
Typical overall average mass spectra obtained from cross sections of raw and fried Bombyx batryticatus by MALDI-TOF-MS in a positive mode. **(A)** Raw Bombyx batryticatus (red). **(B)** Fried Bombyx batryticatus (black).

In order to comprehensively, systematically and accurately identify the various types of active ingredients in raw Bombyx batryticatus and fried Bombyx batryticatus, we analyzed the characteristic ion fragmentation information of the standards, inferring the fragmentation patterns and suitable ionization conditions applicable to the ions of different components. Finally, the positive mode was adopted in the ranges of m/z 100-400 and m/z 400-800 for higher intensities and clearer fragmentation patterns to identify the ionic structure of different components. The representative overall average mass spectra are shown in **Figure 2**. There were 9 amino acid peaks and 12 peptide peaks identified from the transverse sections of Bombyx batryticatus by MS/MS fragmentation patterns of reference standards in conjunction with the precise mass-to-charge ratios, isotopic peaks, data from various literature and other databases (**Supplementary Figure 1**). We detected three main ionic adduct forms, i.e., [M+H]^+^, [M+Na]^+^, and [M+K]^+^. Most amino acids had all been identified as [M+H]^+^ form ions, while a large number of peptides were revealed to be sodium or potassium adducts (**Table 1**). Notably, owing to the extreme degradation and transformation of certain active ingredients in high-temperature environments, the contents of most active ingredients in fried Bombyx batryticatus, with the exception of some specific ingredients, were drastically reduced, although the varieties of compounds contained were essentially identical.

**Table 1:** Amino acids and peptides detected in Bombyx batryticatus by MALDI-TOF-MSI and confirmed by MALDI-TOF-MS/MS.

Compound type		Identify		Molecular formula	Ion information		
					Adduct	m/z	Fragmentations
Amino acid		L-glutamic acid		C_5_H_9_NO_4_	[M+K]^+^	186.02	131.05, 130.05, 102.06 and 84.04
		L-proline		C_5_H_9_NO_2_	[M+H]^+^	116.07	70.07 and 68.05
		L-arginine		C_6_H_14_N_4_O_2_	[M+H]^+^	175.12	158.09, 116.07 and 130.10
		L-tyrosine		C_9_H_11_NO_3_	[M+H]^+^	182.08	165.06, 147.04, 136.08 and 123.04
		L-lysine		C_6_H_14_N_2_O_2_	[M+H]^+^	147.11	130.09, 101.11, 85.09 and 84.08
		Homoarginine		C_7_H_16_N_4_O_2_	[M+H]^+^	189.14	187.40, 145.16, 151.24, 170.22 and 126.91
		γ-aminobutyric acid		C_4_H_9_NO_2_	[M+H]^+^	104.07	103.00, 83.92, 101.32, 58.51 and 73.21
		L-valine		C_5_H_11_NO_2_	[M+H]^+^	118.09	72.08, 59.05, 56.05 and 53.04
		Phosphatidylserine		C_42_H_82_NO_10_P	[M+Na]^+^	814.56	153.00, 281.25, 417.24, 773.53 and 774.54
Peptide		Beauvericin		C_45_H_57_N_3_O_9_	[M+H]^+^	784.42	541.29, 362.19, 262.14 and 234.15
		Beauvericin A		C_46_H_59_N_3_O_9_	[M+H]^+^	798.43	645.32, 545.26, 384.18 and 244.13
		Tenellin		C_21_H_23_NO_5_	[M+H]^+^	370.17	_
		Bassianolide		C_48_H_84_N_4_O_12_	[M+K]^+^	947.57	682.47, 455.31, 328.21 and 228.16
		Beauverolide A		C_30_H_47_N_3_O_5_	[M+Na]^+^	552.34	384.18, 266.13, 244.13 and 131.10
		Beauverolide B		C_31_H_49_N_3_O_5_	[M+Na]^+^	566.36	_
		Beauverolide C		C_35_H_49_N_3_O_5_	[M+H]^+^	592.38	_
		Beauveriolide I		C_27_H_41_N_3_O_5_	[M+K]^+^	526.28	_
		Cyclo(D)-Pro-(D)-Val		C_10_H_16_O_2_N_2_	[M+K]^+^	235.08	_
		Cyclo-(Ala-Pro)		C_8_H_12_O_2_N_2_	[M+H]^+^	169.10	_
		Cyclo(Tyr-Val)		C_14_H_18_N_2_O_3_	[M+K]^+^	301.10	235.32, 190.26, 136.10 and 107.18
		Cyclo(Ala-Tyr)		C_12_H_14_N_2_O_3_	[M+Na]^+^	257.10	129.00, 136.00, 162.00, 207.00 and 235.00

### Multivariate statistical analysis of protein-related metabolites in raw Bombyx batryticatus and fried Bombyx batryticatus

**Figure 3:**
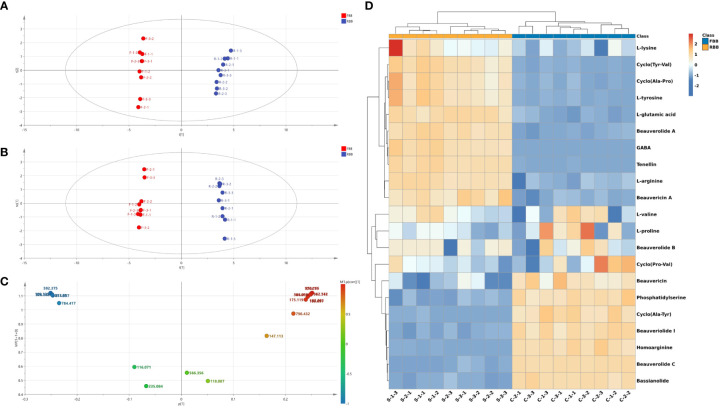
Multivariate statistical analysis of MALDI-TOF-MS data from raw and fried Bombyx batryticatus samples. **(A)** PCA score plot. **(B)** OPLS-DA score plot. **(C)** VIP diagram. **(D)** HCA heatmap.

The raw profile data of endogenous compounds detected were preprocessed including data filtering, area normalization, peak extraction and deconvolution *via* Bruker FlexAnalysis 3.4 software. After the standardization processing, the processed data were loaded into SIMCA-P 14 for further multivariate statistical analysis. Various samples of raw Bombyx batryticatus and fried Bombyx batryticatus were clearly separated into two unique clusters in the principal component analysis (PCA) score plots, indicating substantial distinction between the two treatment groups (**Figure 3A**). The R^2^X (0.848) and Q^2^ (0.691) values of the established PCA model demonstrated its superior fitness and prediction.

OPLS-DA was used to detect characteristic components that cause the differences in the product quality (Gennebäck et al., 2013). The OPLS-DA model clearly distinguished samples of raw Bombyx batryticatus and fried Bombyx batryticatus (**Figure 3B**). The cumulative statistic R^2^X (0.768), model interpretation rate parameter R^2^Y (0.993), and prediction ability parameter Q^2^ (0.985) of the fitted model were all higher than 0.5, indicating that the established model is stable and reliable, and the prediction ability is strong. In addition, OPLS-DA model verification (permutation test n=200) was conducted, resulting in R^2^ of 0.278 and Q^2^ of -0.639 (**Supplementary Figure 2**). The values of R^2^ and Q^2^ were higher than those on the left side, and Q^2^ intersected with the Y-axis on the negative half-axis, indicating that the model is reliable without any overfitting phenomenon.

Variable importance projection (VIP) can reflect the degree of contribution of ions to the model classification. Therefore, differential marker components of amino acids and peptides from raw Bombyx batryticatus and fried Bombyx batryticatus were screened with VIP > 1 as a criterion for differential substances. The results for VIP > 1 included [homoarginine+H]^+^ (m/z: 189.14, VIP=1.16), [beauverolide C acid+H]^+^ (m/z: 592.38, VIP=1.16), [cyclo(Tyr-Val)+K]^+^ (m/z: 301.10, VIP=1.16), [γ-aminobutyric acid+H]^+^ (m/z: 104.07, VIP=1.16), [bassianolide+K]^+^ (m/z: 947.57, VIP=1.16), [L-tyrosine+H]^+^ (m/z:182.08, VIP=1.15), [phosphatidylserine+Na]^+^ (m/z: 814.56, VIP=1.15), [cyclo-(Ala-Pro)+H]^+^ (m/z: 169.10, VIP=1.15), [beauverolide A+Na]^+^ (m/z: 552.34, VIP=1.11), [tenellin+H]^+^ (m/z: 370.17, VIP=1.10), [cyclo(Ala-Tyr)+Na]^+^ (m/z: 257.10, VIP=1.10), [L-arginine+H]^+^ (m/z: 175.12, VIP=1.08), [beauveriolide I+K]^+^ (m/z: 526.28, VIP=1.08), [L-glutamic acid+K]^+^ (m/z: 186.02, VIP=1.05), and [beauvericin+H]^+^ (m/z: 784.42, VIP=1.05) (**Figure 3C**). These ions were the major markers of raw Bombyx batryticatus and fried Bombyx batryticatus.

To reveal metabolic changes between raw Bombyx batryticatus and fried Bombyx batryticatus, a total of 21 protein-related metabolites were visualized in hierarchical clustering analysis (HCA). All data mean values are centralized before analysis, clustering by Euclidean rank and measuring distance by mean link. As shown in **Figure 3D**, each small rectangle in the heat map represents a differentiator, and the content of each metabolite is represented by a standardized range, with darker colors indicating higher metabolite levels. The cluster analysis plot of Bombyx batryticatus samples obviously indicated a division into two sub-groups, a result consistent with the previous PCA and OPLS-DA analyses.

### Spatial distribution of characteristic protein-related metabolites in raw Bombyx batryticatus and fried Bombyx batryticatus

**Figure 4:**
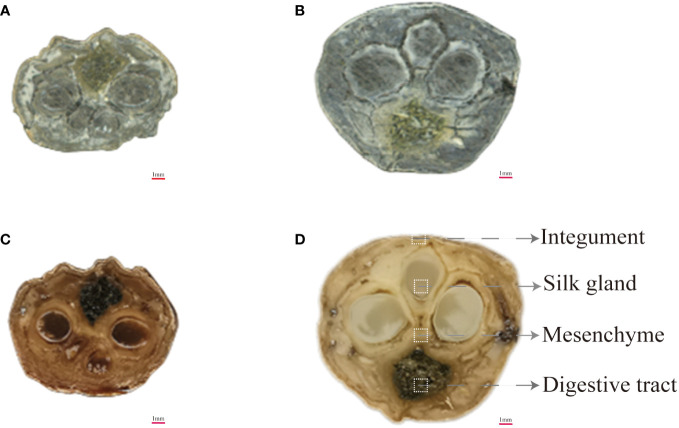
Cross-sectional optical images of raw and fried Bombyx batryticatus. **(A, B)** RBB. **(C, D)** FBB.

MALDI-MSI is an advanced technique to unravel the spatiotemporal variations of the metabolic network of endogenous active ingredients in various tissues and layers of Bombyx batryticatus during the stir-frying process. To further investigate the spatial localization of the diverse metabolic pathways of the bioactive constituents in Bombyx batryticatus, the distribution of signature metabolites of proteins, i.e., peptides and amino acids, was explored in the various tissues. Histologically, the anatomical structure of Bombyx batryticatus was separated into silk gland, digestive tract, mesenchyme and integument from inside to outside (**Figure 4**).

**Figure 5:**
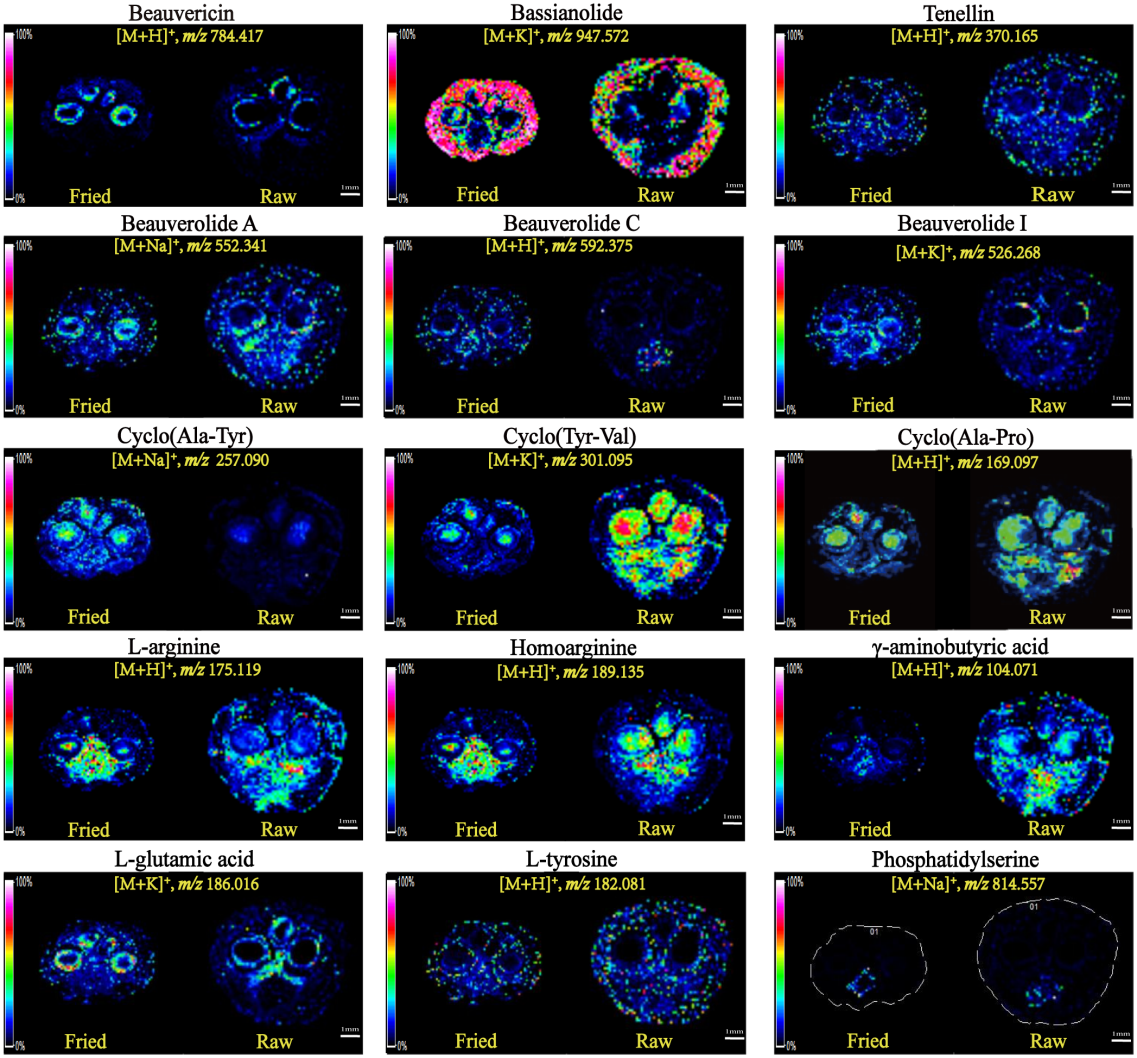
Mass spectrometry images of the ions of representative protein-related metabolites in the tissue sections of raw and fried Bombyx batryticatus.

Among all the compounds, the contents of peptides and amino acids accounted for the highest proportion, which existed in a variety of forms. **Figure 5** illustrates the spatial distribution features of beauvericin (m/z 784.42) and bassianolide (m/z 947.57), two types of ring-shaped polypeptides, which were detected as the [M+H]^+^ and [M+K]^+^ ionic modes. Although they were both cyclic multi-peptide analogs, their distribution patterns were remarkably divergent. The former was more plentiful in the peripheral zone of the silk gland and the latter was more abundant in the epidermis layer of integument. Beauverolide was a large class of cyclic tricarboxylic acid peptide analogs with a variety of subtypes, such as beauverolide A, beauverolide C and beauverolide I. As determined from the ion signal distribution graphs in **Figure 5**, the peripheral tissue of the silk gland, mesenchyme and the digestive tract were the dominant concentration region for beauverolide A in the form of sodium adducts (m/z 552.34), beauverolide C in the form of proton adducts (m/z 592.38) and beauverolide I in the form of potassium adducts (m/z 526.28). It was evident from **Figure 5** that the cyclic dipeptides cyclo(Ala-Tyr) (m/z 257.10), cyclo(Tyr-Val) (m/z 301.10) and cyclo(Ala-Pro) (m/z 169.10) were distributed in the silk gland, digestive tract and mesenchyme region primarily in the form of sodium, potassium and proton adducts, respectively. Notably, the distribution pattern of the proton adducts of tenellin (m/z 370.17) was accumulated in the area corresponding to almost all mesenchyme layers of Bombyx batryticatus. As a consequence of high-temperature stir-frying, the corresponding distribution areas of some certain cyclic dipeptides, such as cyclo(Tyr-Val), cyclo(Ala-Pro), beauverolide A, and tenellin, were reduced to a certain extent, accompanied by a dramatic decrease in the intensity of the detected ion signals. On the contrary, it was noteworthy that the abundance and distribution range of cyclo(Ala-Tyr), beauvericin, bassianolide, beauverolide C, and beauverolide I significantly increased in their respective regions (**Table 2**), which might imply that the latter was more thermally stable.

**Table 2:** Potential markers for the differentiation of raw Bombyx batryticatus and fried Bombyx batryticatus with their relative intensities and localization.

No	Compound	Chemical class	m/z	Existence in raw Bombyx batryticatus		Existence in fried Bombyx batryticatus	
				Intensities	Localization	Intensities	Localization
1	[Beauvericin+H]^+^	Peptides	784.42	31383.56 ± 1931.12	silk gland	39474.44 ± 1122.59	Silk gland
2	[Bassianolide+K]^+^	Peptides	947.57	165276.22 ± 3439.49	integument	222187.22 ± 2923.05	Integument
3	[Tenellin+H]^+^	Peptides	370.17	1595.33 ± 72.81	mesenchyme	374.22 ± 20.61	Mesenchyme
4	[Beauverolide A+Na]^+^	Peptides	552.34	1076.44 ± 47.65	mesenchyme, digestive tract	475.78 ± 28.69	Mesenchyme, Silk gland
5	[Beauverolide C+H]^+^	Peptides	592.38	485.44 ± 64.85	digestive tract	1764.33 ± 28.01	Mesenchyme
6	[Beauveriolide I+K]^+^	Peptides	526.28	431.67 ± 12.97	mesenchyme	647.89 ± 22.03	Mesenchyme
7	[Cyclo(Ala-Tyr)+Na]^+^	Peptides	257.10	490.89 ± 34.28	silk gland	1641.11 ± 158.7	Silk gland
8	[Cyclo(Tyr-Val)+K]^+^	Peptides	301.10	36395.67 ± 3488.47	silk gland, digestive tract	11948.11 ± 677.5	Silk gland
9	[Cyclo(Ala-Pro)+H]^+^	Peptides	169.10	71155.78 ± 7863.57	silk gland, digestive tract	33003 ± 2057.84	Silk gland
10	[L-arginine+H]^+^	Amino acids	175.12	17002.56 ± 258.74	silk gland, digestive tract	13264.11 ± 804.32	Digestive tract
11	[Homoarginine+H]^+^	Amino acids	189.14	34114.78 ± 2136.17	digestive tract	332016.89 ± 26292.6	Silk gland, Digestive tract
12	[γ-aminobutyric acid+H]^+^	Amino acids	104.07	135363.11 ± 8532.19	silk gland, digestive tract	8355.89 ± 235.28	Digestive tract
13	[L-glutamic acid+K]^+^	Amino acids	186.02	2053 ± 28.22	mesenchyme	1704.67 ± 25.79	Mesenchyme
14	[L-tyrosine+H]^+^	Amino acids	182.08	951.78 ± 119.21	mesenchyme	358.22 ± 19.33	Mesenchyme
15	[Phosphatidylserine+Na]^+^	Amino acids	814.56	1047.89 ± 63.83	digestive tract	1724.44 ± 77.49	Digestive tract

Amino acids, the most widely distributed chemical compounds in Bombyx batryticatus, were virtually present as proton adducts and exhibited a variable distribution in multiple areas. γ-aminobutyric acid (GABA), as a small non-protein amino acid, was an important inhibitory neurotransmitter of the central nervous system that existed widely in vertebrates, plants and microorganisms (Cifelli et al., 2021). L-arginine (m/z 175.12), homoarginine (m/z 189.14) and GABA (m/z 104.07) were more concentrated in the medial part of the silk gland and the digestive tract (**Figure 5**). In contrast, it was noted that a kind of amino acid was more plentiful in the peripheral region of the silk gland (**Figure 5**), which was ultimately characterized as L-glutamic acid (m/z 186.02), i.e., a kind of nonessential amino acid possessing the effect of treating hepatic coma. The distribution pattern of the proton adducts of L-tyrosine (m/z 182.08) was completely different from the above-mentioned localization of amino acids and accumulated at the area corresponding to almost all regions except the silk gland. Phosphatidylserine (m/z 814.56) appeared to have a clear tendency to distribute only in the central area of the digestive tract (**Figure 5**). Furthermore, it was noteworthy that the distribution areas of a considerable percentage of amino acids were shrunk to a large extent along with the content levels decreased significantly, whereas those of homoarginine increased substantially through the high-temperature processing in contrast (**Table 2**).

## Discussion

By integrating factors such as tissue integrity, data accuracy and detection sensitivity, we eventually set the section thickness of the tissue at 20 μm and the temperature at -25°. Further, in the pre-experiments for the extraction of active ingredients of Bombyx batryticatus we observed that more ionic peaks could be detected in the positive mode by spraying with 2-MBT as the matrix compared to the negative mode, possibly related to the spatial structure and ionization mode of the compounds. Therefore, we determined to choose the positive mode in our experiments.

The body of the authentic Bombyx batryticatus was white, straight, stiff, crisp, etc., among which the key characters that determined the quality of Bombyx batryticatus were the traits of silk gland in the cross section of Bombyx batryticatus after fracture. The transverse section of Bombyx batryticatus with the best curative properties was described as black, full and shiny like mirrors (Hu et al, 2017). By virtue of the *in situ* spatial distribution of 15 signature peptides and amino acids, we found that silk gland tissue was the enrichment region for the majority of active ingredients, demonstrating the spatial localization advantage of MSI over traditional analysis techniques. In the field of Chinese medicine research and development, silk gland could be regarded as the primary region for extracting the effective components of Bombyx batryticatus, so as to improve potency and reduce side effects.

Beauvericin is a cyclic tricarboxylic acid peptide, the essentially secondary metabolite secreted by *Beauveria bassiana* during the growing process as one of the most crucial active ingredients of the Bombyx batryticatus (Chen et al., 2019). We detected various types of beauvericin and its derivatives, such as beauvericin A, bassianolide and beauverolide A, which had pharmacological properties including antitumor (Zhan et al., 2007; Jiang et al., 2014; Wu et al., 2017), antibacterial (Kouri et al., 2005; Mei et al., 2009), antiviral, and anticonvulsant effects (Jiang et al., 2023). Cyclodipeptide is formed by the internal cyclization of two amino acids, with 2,5-diketopiperazine or 2,5-dioxypiperazine as its parent nucleus (Wei, 2020). There were several types of cyclic dipeptides identified by MALDI-MSI, such as cyclo(Tyr-Val) and cyclo(Ala-Pro), with the bioactivities of anti-tumor, neuroprotective, immune and metabolic regulatory, and anti-inflammatory effects (Qiang and Mei, 2014). It was revealed that some certain peptides and amino acids in stir-fried Bombyx batryticatus were elevated in specific regions by MSI, which might be attributed to the local degradation effect of proteins. Notably, we observed that the apparent increase in the abundance of beauvericin and bassianolide was accompanied by dramatic descents of cyclo(Tyr-Val) and cyclo(Ala-Pro). Such a tendency of variation in the content of peptides might greatly indicate the enhancement of antitumor, antiviral and anticonvulsant effects, as well as the decrease of antioxidant, neuroprotective, immune and metabolic regulatory activities.

GABA is an important active ingredient in mulberry leaves. It was reported that the intake of certain amounts of GABA had physiological effects, such as improving the quality of sleep, reducing toxic impairment of hepatocytes, and lowering blood pressure (Nakamura et al., 2011). Arginine is involved in the ornithine cycle in the body, which facilitates the conversion of ammonia produced in the body into non-toxic urea for excretion, thereby reducing the blood ammonia concentration and correcting the acid-base balance in hepatic encephalopathy (Mangoni et al., 2019). Furthermore, homoarginine is a naturally occurring, non-protein-derived, cationic amino acid, which increases the availability of nitric oxide (NO), affecting endothelial function (Rodionov et al., 2021). Elevated levels of homoarginine could exert positive effects that were associated with cardiovascular protection, in terms of strengthening endothelial function, suppressing platelet aggregation and boosting insulin secretion (Kathrin et al., 2019). The results of our study demonstrated that significant decreases in arginine and GABA levels were accompanied by increased homoarginine content during the stir-frying process. The fried Bombyx batryticatus may not be as effective as raw Bombyx batryticatus in treating liver disease and neurotrophic effects, while it was more valuable in reducing the risk of cardiovascular disease (Kong et al., 2014; Zhao et al., 2018).

In recent years, it had been reported that the consumption of raw Bombyx batryticatus could sometimes trigger adverse reactions, probably due to the entry of foreign proteins into the body, which could lead to allergy and intense multi-organ syndromes through the immune mechanism, manifesting as respiratory obstruction, circulatory symptoms and abdominal pain as well as other type I allergic reactions. The allergen was also cross-reactive with the central nervous system, resulting in metabolic disorders and dysfunction of the central nervous system (Tu and Yu, 2012). Therefore, it was confirmed that the Bombyx batryticatus should be stir-fried with wheat bran before being applied to therapeutic purposes on account of the clinical experience from ancient Chinese medicine practitioners. As a matter of fact, this empirical medical theory was not fully accepted by the western medicine. Notably, it could be demonstrated from the rigorous data obtained in our study that fried Bombyx batryticatus had the following advantages over raw Bombyx batryticatus with reference to the relevant literature.

Above all, during the process of high-temperature frying, the energy absorbed by the protein exceeded the intermolecular interaction force, leading to the destruction of the spatial conformation in protein, followed by the rupture of the peptide chain, thus partially denaturing and inactivating the protein (Lin et al., 2020). The reduction of the animal protein and peptides in the fried Bombyx batryticatus moderated the irritation of the medicine applied to the patient, which was in accordance with the principles of processing (Ma et al., 2015). In addition, due to the breakage of the peptide bond by thermal oscillation during the stir-frying process, small molecule oligopeptides could be produced for oral absorption, solving the problem of low absorption rate of natural oligopeptides and the difficulty of industrialization, and providing an experimental basis for the development and utilization of the Bombyx batryticatus resources (Li et al., 2019).

Furthermore, the interaction between the excipients and the drug existed in the container because of substantial addition of wheat bran during the process of concoction. The surface of wheat bran was loose and porous with a higher surface area, reinforcing the adsorption activity. Meanwhile, wheat bran was rich in dietary fiber possessing an anti-oxidant effect. After being fried, there was plenty of dietary fiber remaining on the surface of the Bombyx batryticatus, which inhibited the biosynthesis of fungal toxins (Zhao et al., 2010). As a consequence of Maillard reaction on the Bombyx batryticatus during the stir-frying process, the epidermis of Bombyx batryticatus became golden in color with an aromatic smell, which promoted the digestive function and improves the therapy compliance of patients when taking medicine (Hu et al., 2016; Li et al., 2021b).

## Conclusion

In this study, a high-resolution MALDI-MSI technique was applied to elucidate the *in situ* distribution of protein-related metabolites in the cross sections of raw and fried Bombyx batryticatus. Ultimately, 15 processing-related markers were screened and visualized in combination with OPLS-DA, which may have a significant impact on the research of the holistic chemical transformation mechanism of Bombyx batryticatus before and after frying with wheat bran.

## Data availability statement

The original contributions presented in the study are included in the article/**Supplementary Material**. Further inquiries can be directed to the corresponding authors.

## Author contributions

PL and J-MW performed the experiments and preparation of the draft manuscript. H-CG, M-WZ, and Y-XS analyzed the data. HG and X-HD prepared figures and tables. Y-PY project conceptualization, conceived, and designed the experiments. Y-GZ conceived and designed the experiments, and reviewed drafts of the paper. All authors contributed to the article and approved the submitted version.
