# Supplementary material for: In situ detection and mass spectrometry imaging of protein-related metabolites in Bombyx batryticatus before and after frying with wheat bran
Source: Front Plant Sci. 2023 Apr 6;14:1144556. doi: 10.3389/fpls.2023.1144556 (PMC10117890; doi:10.3389/fpls.2023.1144556)
Supplement: Supplementary file 1 [file DataSheet_1.pdf]

Supplementary Material

In Situ Detection and Imaging of the Bombyx Batryticatus Proteins before and after Frying with Wheat Bran Using MALDI-MSI

Pai Liu†, Jie-Min Wang†, Hao-Chuan Guo, Meng-Wei Zhao, Yong-Xing Song, Hui Guo, Xu-Hong Duan, Yu-Ping Yan*, Yu-Guang Zheng*

[*] **Correspondence:** Yu-Ping Yan: yanyuping2008@126.com; Yu-Guang Zheng: zyg314@163.com

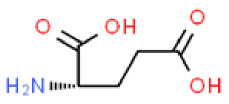

L-glutamic acid

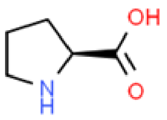

L-proline

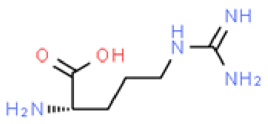

L-arginine

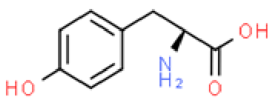

L-tyrosine

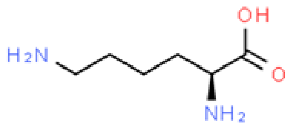

L-lysine

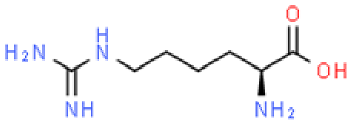

Homoarginine

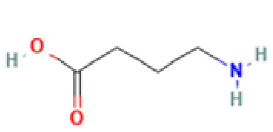

γ-aminobutyric acid

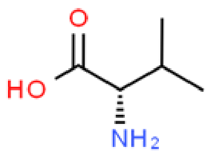

L-valine

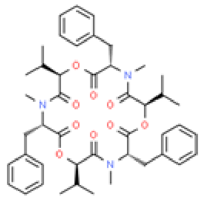

Beauvericin

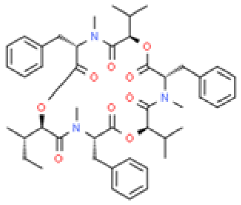

Beauvericin A

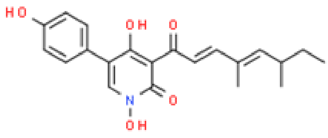

Tenellin

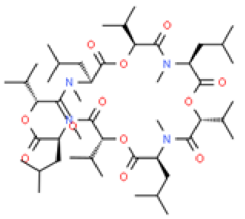

Bassianolide

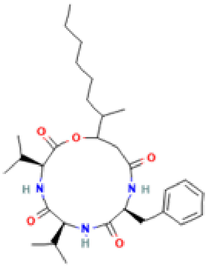

Beauverolide A

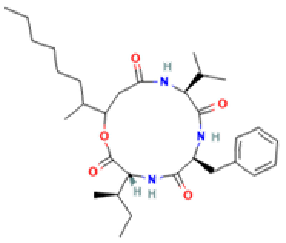

Beauverolide B

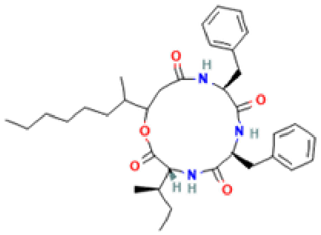

Beauverolide C acid

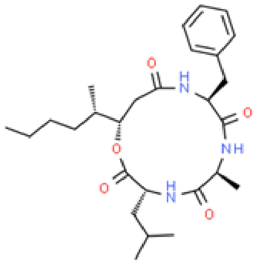

Beauveriolide I

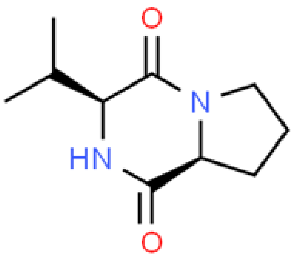

Cyclo(D)-Pro-(D)-Val

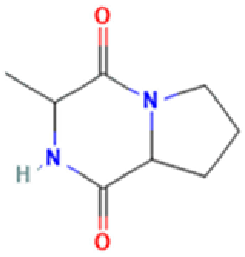

Cyclo-(Ala-Pro)

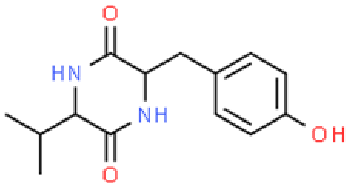

Cyclo(Tyr-Val)

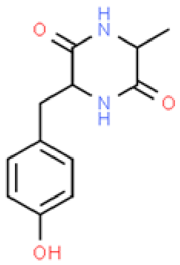

Cyclo(Ala-Tyr)

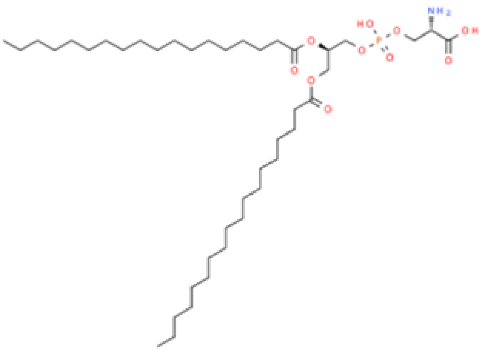

Phosphatidylserine

Supplementary Figure 1. Chemical structure of 21 compounds in Bombyx Batryticatus.

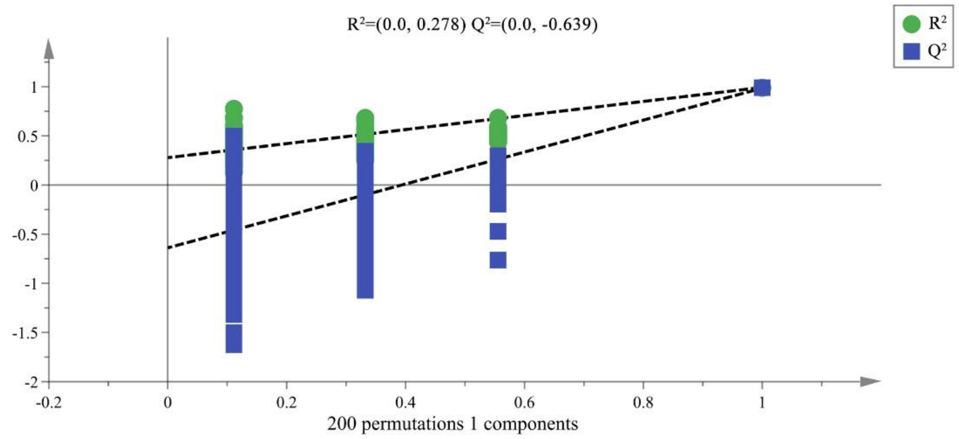

Supplementary Figure 2. Permutation plot of OPLS-DA.

Supplementary Table 1. Compound name, chemical class, and supplier of the reference standards.

Compound name	Chemical class	Supplier
γ-aminobutyric acid	Amino acids	National Institutes for Food and Drug Control (Beijing, China), NIFDC for short
L-lysine	Amino acids	NIFDC
L-proline	Amino acids	NIFDC
L-valine	Amino acids	NIFDC
L-arginine	Amino acids	NIFDC
L-tyrosine	Amino acids	NIFDC
L-glutamic acid	Amino acids	NIFDC
Homoarginine	Amino acids	NIFDC
Beauvericin	Peptides	NIFDC
Bassianolide	Peptides	NIFDC
Adenine	Nucleosides	NIFDC
Hypoxanthine	Nucleosides	NIFDC
Xanthine	Nucleosides	NIFDC
Thymidine	Nucleosides	NIFDC
Adenosine	Nucleosides	NIFDC
Hypoxanthine	Nucleosides	NIFDC
Xanthine	Nucleosides	NIFDC
Citric acid	Organic acid	NIFDC
Choline	Organic base	NIFDC
Quercetin	Flavonoids	NIFDC
Kaempferol	Flavonoids	NIFDC
Betaine	Alkaloids	NIFDC
L-Carnitine	Alkaloids	NIFDC
Stearic acid	Fatty acids	NIFDC
Palmitic acid	Fatty acids	NIFDC
β-Sitosterol	Steroids	NIFDC

Supplementary Table 2. Student's t-test analysis of the differential metabolites.

m/z	T-value	P-value	-log10P	FDR
104.071	-28.66	3.5191E-15	14.454	1.2317E-14
147.113	-3.3031	0.0044899	2.3478	0.0055464
169.097	-13.276	4.6966E-10	9.3282	7.045E-10
175.119	-12.515	1.1168E-09	8.952	1.5636E-09
182.081	-13.901	2.3781E-10	9.6238	3.8415E-10
186.016	-25.775	1.8569E-14	13.731	5.5707E-14
189.135	69.774	2.6074E-21	20.584	5.4756E-20
257.09	20.038	9.2912E-13	12.032	2.168E-12
301.095	-19.458	1.4606E-12	11.835	3.0673E-12
370.165	-45.641	2.2456E-18	17.649	1.5719E-17
526.268	23.921	5.9596E-14	13.225	1.5644E-13
552.341	-30.544	1.2928E-15	14.888	5.4298E-15
592.375	51.21	3.6009E-19	18.444	3.7809E-18
784.417	10.245	1.9591E-08	7.7079	2.5714E-08
798.432	-14.78	9.5345E-11	10.021	1.6685E-10
814.557	19.062	2.0049E-12	11.698	3.8276E-12
947.572	35.662	1.1216E-16	15.95	5.8883E-16
